# Genetic and Linguistic Coevolution in Northern Island Melanesia

**DOI:** 10.1371/journal.pgen.1000239

**Published:** 2008-10-31

**Authors:** Keith Hunley, Michael Dunn, Eva Lindström, Ger Reesink, Angela Terrill, Meghan E. Healy, George Koki, Françoise R. Friedlaender, Jonathan S. Friedlaender

**Affiliations:** 1 Department of Anthropology, University of New Mexico, Albuquerque, New Mexico, United States of America; 2 Centre for Language Studies, Radboud University, Nijmegen, The Netherlands; 3 Max Planck Institute for Psycholinguistics, Nijmegen, The Netherlands; 4 Department of Linguistics, Stockholm University, Stockholm, Sweden; 5 Human Genetics, Institute for Medical Research, Goroka, Papua New Guinea; 6 Independent Researcher, Sharon, Connecticut, United States of America; 7 Department of Anthropology, Temple University, Philadelphia, Pennsylvania, United States of America; University of Chicago, United States of America

## Abstract

Recent studies have detailed a remarkable degree of genetic and linguistic diversity in Northern Island Melanesia. Here we utilize that diversity to examine two models of genetic and linguistic coevolution. The first model predicts that genetic and linguistic correspondences formed following population splits and isolation at the time of early range expansions into the region. The second is analogous to the genetic model of isolation by distance, and it predicts that genetic and linguistic correspondences formed through continuing genetic and linguistic exchange between neighboring populations. We tested the predictions of the two models by comparing observed and simulated patterns of genetic variation, genetic and linguistic trees, and matrices of genetic, linguistic, and geographic distances. The data consist of 751 autosomal microsatellites and 108 structural linguistic features collected from 33 Northern Island Melanesian populations. The results of the tests indicate that linguistic and genetic exchange have erased any evidence of a splitting and isolation process that might have occurred early in the settlement history of the region. The correlation patterns are also inconsistent with the predictions of the isolation by distance coevolutionary process in the larger Northern Island Melanesian region, but there is strong evidence for the process in the rugged interior of the largest island in the region (New Britain). There we found some of the strongest recorded correlations between genetic, linguistic, and geographic distances. We also found that, throughout the region, linguistic features have generally been less likely to diffuse across population boundaries than genes. The results from our study, based on exceptionally fine-grained data, show that local genetic and linguistic exchange are likely to obscure evidence of the early history of a region, and that language barriers do not particularly hinder genetic exchange. In contrast, global patterns may emphasize more ancient demographic events, including population splits associated with the early colonization of major world regions.

## Author Summary

The coevolution of genes and languages has been a subject of enduring interest among geneticists and linguists. Progress has been limited by the available data and by the methods employed to compare patterns of genetic and linguistic variation. Here, we use high-quality data and novel methods to test two models of genetic and linguistic coevolution in Northern Island Melanesia, a region known for its complex history and remarkable biological and linguistic diversity. The first model predicts that congruent genetic and linguistic trees formed following serial population splits and isolation that occurred early in the settlement history of the region. The second model emphasizes the role of post-settlement exchange among neighboring groups in determining genetic and linguistic affinities. We rejected both models for the larger region, but found strong evidence for the post-settlement exchange model in the rugged interior of its largest island, where people have maintained close ties to their ancestral lands. The exchange (particularly genetic exchange) has obscured but not completely erased signals of early migrations into Island Melanesia, and such exchange has probably obscured early prehistory within other regions. In contrast, local exchange is less likely to have obscured evidence of population history at larger geographic scales.

## Introduction

In *On the Origin of Species* [1] and *The Descent of Man* [2], Darwin suggested that patterns of global biological and linguistic variation might correspond because of their parallel evolution in isolated human groups. Recently, Cavalli-Sforza and colleagues [3]–[5] described a more formal version of this process in which congruent genetic and linguistic trees form as a result of serial population splits and isolation that occur during range expansions into new territories.

Anthropologists [e.g., 6],[7] have long been skeptical of this “branching” model of genetic and linguistic coevolution, being wary of conflating biological evolution and cultural change, and because any tight link between the two forms of variation could only occur if past human populations remained isolated following the splits. While it is conceivable that they did so for short periods as they expanded to fill unoccupied regions [8], the prolonged isolation required for congruent evolution seems unlikely.

Genetic and linguistic correspondence may also form through a process that is analogous to the genetic model of isolation by distance [9]–[11]. In this process, populations are arrayed evenly over a geographic landscape and neighboring populations exchange both genetic and linguistic features. Genetic and linguistic features may move independently of one another, in which case a correlation will form between genetic and linguistic distances that is purely the result of the underlying correlation of both with geographic distance [3],[12],[13]. Genetic and linguistic features may also move between groups together, in which case their underlying correlation will be independent of geographic distance [13].

Earlier studies have not provided convincing support for either the branching or isolation by distance processes for gene-language coevolution. Cavalli-Sforza and colleagues [5] found some congruence between global gene and language trees, but their informal method of tree comparison was subsequently challenged [14]. With a more formal test, Hunley and colleagues rejected the branching model in Native North America [15] and Native South America [16], though they found some superficial congruence between gene and language trees. The isolation by distance coevolution process has seldom been explicitly tested, but studies in several world regions have either failed to identify genetic and linguistic correlations of any kind or have identified only weak correlations [13], [17]–[25].

Several factors may account for the lack of evidence for gene-language coevolution. First, genes and languages may disperse in very different ways simply because biological transmission is solely vertical but linguistic transmission is both vertical and horizontal [7],[26]. The differing modes of biological vs. linguistic transmission might, in the long term, disrupt correspondences that initially formed through the branching process. Second, differing rates of neutral genetic and linguistic evolution, or differing selective pressures, may prevent the formation of stable genetic and linguistic correspondences [3],[19],[27],[28]. Third, the large geographic scale of many of these studies might prevent the detection of linguistic and genetic correspondences that form at more local levels [16],[29]. Finally, gene-language correspondences could be blurred by the combination of continual group movements and inter-group exchange.

The lack of strong support for coevolution may also reflect deficiencies in the methods used to examine linguistic variation. Many studies employ controversial language classifications estimated from cognate data [30]–[32] and estimate linguistic distances simply by counting nodes in these classifications [16], [33]–[36]. Even if a classification is correct, node counting may produce particularly inaccurate distances for long-separated languages [4],[37].

In this study, we compared detailed genetic and linguistic patterns from data collected across a set of particularly diverse populations in the Southwest Pacific. To construct a linguistic classification and estimate linguistic distances, we used data from over 100 structural linguistic features (i.e., aspects of sound systems and grammar) that may avoid some of the limitations associated with cognate data [37]–[39]. These linguistic data, and high-quality autosomal microsatellite data, were used to test predictions of the two coevolutionary models.

The datasets come from Northern Island Melanesia, a region well-known for its complex history and remarkable biological and linguistic diversity [40]. The earliest inhabitants of the region arrived at least 40,000 years ago and are thought to have diversified in place in relative isolation from the rest of humanity for the following 30,000 years [41], but there is clear evidence of at least one additional population movement into the region from farther west about 3,300 years ago [42]. The region is geographically complex, with a set of neighboring islands varying in size and ruggedness. As a result, it is a particularly informative region to analyze factors mediating or inhibiting the formation of genetic and linguistic correspondences.

### Background

**Figure 1:**
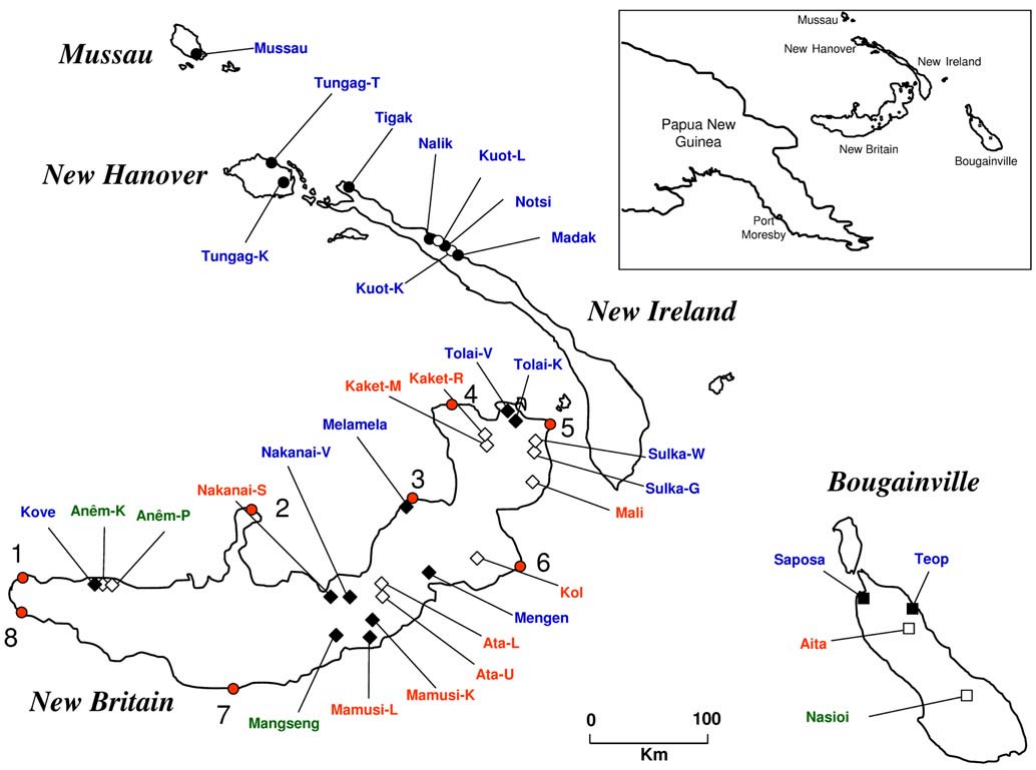
Map and population locations. Colors show interior vs. coastal locations: blue for coastal; red for interior; green for intermediate locations. The Nakanai are represented by both a coastal and an interior population. Filled shading vs. open shapes show language affiliation: Oceanic languages are filled; Papuan languages are open. Shapes show island location: diamonds for New Britain; squares for Bougainville; circles for New Ireland, New Hanover, and Mussau. Numbers are waypoints used to estimate geographic distances between populations on New Britain and between populations on New Britain and other islands.

The languages of Northern Island Melanesia (NIM) belong to two major groups: Oceanic and Papuan. Oceanic is a major branch of the widespread Austronesian language family that appeared in the region about 3,300 years ago [43], almost certainly associated with the Lapita cultural complex [42],[44]. In NIM, Oceanic languages are found mainly on the smaller offshore islands and along the coasts of the major islands (see Figure 1), though they are spoken in some large island interiors as well. Our sample includes populations that speak 14 of the more than 150 Oceanic languages spoken in the region today. The Papuan languages are likely descendents of languages spoken by people who began arriving in the region more than 40,000 years ago [38],[45]. As a result of their antiquity, they do not form a coherent language family according to conventional historical linguistic criteria, but are rather a residual category of non-Austronesian languages [37]. The Papuan languages in NIM tend to be restricted to the interior highlands of New Britain and Bougainville (Figure 1). Our sample includes populations that speak 9 of the 20 or so Papuan languages spoken in the region today.

The standard method of constructing the historical relationships between languages, called the Comparative Method, is a tree-building technique that relies on recognizing sets of words in different languages that are related in meaning and form (cognates) and which show regular sound changes (i.e., shared innovations) demonstrating that they derive from a single ancestral language. Because cognates change relatively rapidly, reconstructions using the Comparative Method cannot generally be made beyond 8,000 years [32]. In NIM, the Papuan languages share no clearly related cognates, possibly because they have been isolated from one another for so long, making the Comparative Method inapplicable for examining their relationships [37],[46],[47].

Recently, Dunn and colleagues [37] proposed the use of abstract structural linguistic features to address the time-depth constraint. These features could provide an independent phylogenetic measure, not related to the lexical evidence. Structural features include syntactic patterns such as constituent order in clauses and noun phrases, paradigmatic structures of pronouns, and the structure of verbal morphology [38]. It is an open question whether structural features are in general more resistant to exchange between different languages, but in contrast to cognate data, the Papuan languages of NIM do show some structural similarity, suggesting that, at least in this case, structural features are more stable [37]. However, structural features are not without their problems, including possible non-independence and homoplasy. To examine their utility and consistency for historical linguistic reconstruction, Dunn and colleagues [37] compared an Oceanic language classification constructed with structural data to one constructed using the Comparative Method. The topologies of the two trees were quite similar. Their structural classification of Papuan languages in NIM also captured the geography of the region fairly well, with its major branches representing the languages of different islands and its more terminal branches joining geographic neighbors within islands. These results were confirmed in subsequent analyses [48],[49] and suggest that structural linguistic features may well produce reliable language trees and linguistic distances estimates, at least in NIM.

### Model Predictions

The branching model predicts that the patterns of linguistic and genetic variation will be treelike, so that for our datasets, the Oceanic- and Papuan-speaking populations will cluster on separate branches of the language and genetic trees, and it also predicts that the topologies within the separate Oceanic and Papuan clusters will be similar in both trees. We tested these predictions by comparing simulated and observed patterns of genetic variation and the topologies of gene and language trees.

The isolation by distance model predicts that genetic and linguistic distances will be correlated with one another not because of congruent tree-like evolution but because of ongoing genetic and linguistic exchange between neighboring populations. If genetic and linguistic exchange have occurred independently of one another, the genetic-linguistic distance correlation will lose statistical significance when geographic distance is held constant. If they have moved largely in concert with one another, the genetic-linguistic distance correlation will remain significant when geographic distance is held constant. These predictions were tested using computer simulations, matrix correlation and partial correlation tests, and by examining plots of genetic, linguistic and geographic distances.

## Materials and Methods

### Data

The detailed genetic and linguistic datasets were recently collected from 33 populations located on the major islands of the Bismarck Archipelago and Bougainville in NIM [38],[39],[50] (Figure 1, Table 1). The genetic data consist of 751 autosomal microsatellite loci drawn from Marshfield Screening sets # 16 and # 54, and the loci were typed in 776 individuals. The linguistic data consist of 108 abstract structural features scored as present or absent in 23 Northern Island Melanesian languages. The features provide broad typological coverage of the known linguistic variation of the region and represent features typically described in a published sketch grammar. Three language groups covered in the genetic survey had not been analyzed (see Table 1), and for them, we substituted data from very closely related languages.

**Table 1:** Sample details.

Population	Sampling location	Sample size	Island	Language group	Interior vs. Coast	Lat	Long	Allelic identity
Tigak	Kaplaman	23	New Ireland	Oceanic	Coast	−2.6	150.9	0.306
Nalik	Fatmilak	25	New Ireland	Oceanic	Coast	−3.0	151.5	0.308
Notsi	Amba	25	New Ireland	Oceanic	Coast	−3.1	151.7	0.309
Tungag-T	Tsoi	24	New Hanover	Oceanic	Coast	−2.4	150.4	0.309
Mangseng	Ru	20	New Britain	Oceanic	Intermediate	−5.9	150.7	0.309
Tolai-V	Vunairoto	25	New Britain	Oceanic	Coast	−4.2	152.1	0.309
Kuot-L	Lamalaua	18	New Ireland	Papuan	Coast	−3.0	151.5	0.309
Mussau	Lovarang	24	Mussau	Oceanic	Coast	−1.6	149.7	0.310
Teop^1^	Inivus	24	Bougainville	Oceanic	Coast	−5.9	155.2	0.311
Nakanai-V	Valoka	25	New Britain	Oceanic	Coast	−5.8	150.8	0.312
Tungag-K	Kulingai	24	New Hanover	Oceanic	Coast	−2.6	150.4	0.313
Mengen	Ulamona	24	New Britain	Oceanic	Coast	−5.1	151.4	0.313
Sulka-G	Ganai	24	New Britain	Papuan	Coast	−4.5	152.3	0.313
Saposa	Toruai	25	Bougainville	Oceanic	Coast	−5.6	154.7	0.314
Melamela	Ubili	25	New Britain	Oceanic	Coast	−5.0	151.3	0.315
Kuot-K	Kabil	25	New Ireland	Papuan	Coast	−3.1	151.7	0.316
Madak	Lamasong	24	New Ireland	Oceanic	Coast	−3.1	151.7	0.316
Sulka-W	Watwat	18	New Britain	Papuan	Coast	−4.3	152.3	0.317
Kove	Arumigi	25	New Britain	Oceanic	Coast	−5.5	149.0	0.323
Tolai-K	Kabakada	24	New Britain	Oceanic	Coast	−4.5	152.1	0.324
Anêm-K	Keraiai	22	New Britain	Papuan	Intermediate	−5.5	149.0	0.326
Anêm-P	Purailing	23	New Britain	Papuan	Intermediate	−5.5	149.0	0.330
Kol	Nutuve	21	New Britain	Papuan	Interior	−5.4	151.6	0.331
Ata-L	Lugei	25	New Britain	Papuan	Interior	−5.6	151.0	0.338
Nasioi	Rumba	24	Bougainville	Papuan	Intermediate	−6.5	155.8	0.339
Nakanai-S	Silanga	18	New Britain	Oceanic	Interior	−5.5	150.8	0.343
Mamusi-K^2^	Kisiluvi	25	New Britain	Oceanic	Interior	−5.7	151.1	0.347
Ata-U	Uasilau	25	New Britain	Papuan	Interior	−5.7	151.0	0.350
Mamusi-L^2^	Lingite	25	New Britain	Oceanic	Interior	−5.9	151.1	0.356
Aita^3^	Kukuavo	25	Bougainville	Papuan	Interior	−5.9	155.1	0.362
Kaket-R	Rangulit	22	New Britain	Papuan	Interior	−4.4	151.9	0.377
Kaket-M	Malasait	25	New Britain	Papuan	Interior	−4.5	151.9	0.382
Mali	Marabu	25	New Britain	Papuan	Interior	−4.6	152.3	0.382
Total		776						

1 Saposa was used as a proxy for the Teop language.

2 Uvol was used as a proxy for Mamusi.

3 Rotokas was used as a proxy for Aita.

The population names are linguistically based. Where genetic data were collected from more than one group in a language area, we added a distinguishing letter (e.g., Anêm-K and Anêm-P for the two Anêm-speaking groups from the Keraiai and Purailing areas). Table 1 lists each population name, island, language affiliation, geographic coordinates, genetic sample size and allelic identity (by which the populations are ordered). Because of recent movements, three populations could not be clearly classified as coastal or interior, and they were therefore classified as “intermediate”. The linguistic and genetic data are available from the authors upon request.

### Analytical Methods

Our basic unit of genetic similarity is the allelic identity between individuals, defined as the probability that two alleles of the same locus drawn from two random individuals, either within the same population or from two different populations, are identical [51]. Heat plots were employed to examine the geographic and linguistic patterns of the within- and between-population allelic identities.

#### The branching model

**Figure 2:**
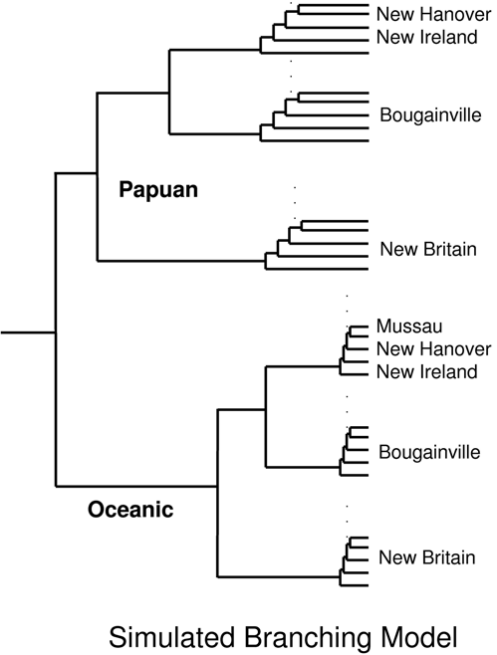
Population history for the branching model simulations. The first division is between Oceanic and Papuan languages, and subsequent splits occur in a nested fashion between and within each island. In the simulations, there is no migration between any populations.

We used coalescent-based computer simulations to construct the predicted pattern of allelic identity variation for the branching model. The simulations are detailed in Text S1. The presumed history of population splits used as the basis for the simulated branching model is shown in Figure 2. The first division is between Oceanic- and Papuan-speaking populations, whose ancestors would have separated long before the initial settlement of NIM and whose descendants would have continued to remain separate according to the branching model. The model also predicts that subsequent splits would have occurred in a nested fashion between and then within each island and that no migration would have occurred between populations.

Trees were constructed with different techniques. The unrooted language tree was constructed from the 108 structural linguistic items across the 23 languages using the Bayesian approach described by Huelsenbeck and Ronquist [52]. The autosomal microsatellite tree (hereafter referred to as the genetic tree) was constructed from a matrix of population pairwise R_ST_ genetic distances [53] using the neighbor joining method [54]. Further details of the tree-building methods are described in Text S1.

To compare the trees formally, a modified version of the Cavalli-Sforza and Piazza [55] test of treeness was used. This method estimates an allelic identity matrix for the language tree (or any other tree) that is as similar to the allelic identity matrix estimated from the microsatellites as possible, given the constraints of the topology of the language tree. The degree of similarity between the “expected” language tree-estimated matrix and the “observed” microsatellite matrix is measured by a likelihood ratio statistic, Λ [55]–[57]. Under the assumption of a large number of independently evolving loci, Λ is distributed as a χ^2^ random variable, with degrees of freedom equal to *s*(*s*+1)/2 minus the number of nodes in the language tree, where *s* is the number of populations. The expected value of Λ is equal to the degrees of freedom if the language tree “fits” perfectly. Further details of the method are provided in Text S1.

To further compare the linguistic and genetic structure, we also fitted the simplest possible tree in which all populations diverged from a common ancestor at the same time in the past. In this tree, there is only one internal node connecting all of the populations. Because the tree contains no internal structure other than this single internal node, it can serve as a baseline against which the fit of the language tree can be compared. The fit of the language tree relative to this baseline tree was estimated with an F-test, 
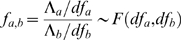
, where Λ_a_ is the likelihood ratio statistic of the baseline tree with *df_a_* degrees of freedom, and Λ_b_ is the likelihood ratio statistic for the language tree. Lewis and Long [58] suggested that this test could be used to compare the fit of any two trees where one is made by adding nodes to the other, as is the case for the language tree relative to the baseline tree. The test is valid under the assumption that, if the Λ values for the two trees are equally inflated relative to the chi-squared distribution, then the inflation factor will cancel in their ratio.

As a third way to evaluate the fit of the language tree to the genetic structure of NIM populations, we estimated the genetic *distances* between populations from the observed allelic identity matrix, then estimated genetic distances from the language tree-expected allelic identity matrix, and finally plotted these two sets of genetic distances against one another. Besides allowing a simple visual comparison of the correspondence between the observed genetic pattern and the predicted pattern for the language tree, the residuals of the plot may be examined to assess the specific causes of any observed lack of correspondence. Genetic distances were estimated from the allelic identities using the formula of Nei [51]: 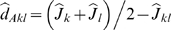, where *J_k_* and *J_l_* are the allelic identities in populations *k* and *l*, and *J_kl_* is the allelic identity between populations *k* and *l*.

#### The isolation by distance model

We used the coalescent-based computer simulations to estimate an allelic identity matrix for the isolation by distance model (see Text S1) and then used heat plots to compare the observed and simulated allelic identity matrices. We also compared matrices of genetic, linguistic and geographic distances between population pairs using matrix correlation and partial correlation tests [59],[60]. The elements of the linguistic distance matrix are the proportion of different features between pairs of languages (the matrix is provided in Text S1). Great circle geographic distances were computed from the geographic coordinates provided in Table 1 using the haversine function [61]. Geographic distances were computed directly between each population pair and also using eight waypoints on the New Britain coast (see Figure 1). The waypoint approach estimated geographic distances between coastal New Britain populations only along the coasts, and between New Britain and the other islands through the northeast coast of New Britain (Figure 1, waypoint 5). The partial correlation tests measured the correlation between genetic and linguistic distances while holding geographic distance constant. Since there is some debate about significance values for partial correlation tests [62]–[64], they should be interpreted cautiously.

## Results

The last column of Table 1 shows that the Oceanic-speaking populations generally have lower allelic identities than the Papuan-speaking populations. The mtDNA and Y-chromosome data in the same populations have a similar pattern [65]–[67], and the mtDNA and Y-chromosome distances are also much higher between Papuan-speaking populations. This was taken to show the primary action of genetic drift in small isolated groups of Papuan speakers that arrived very early in the region. The Oceanic-speaking populations arrived much more recently, lived in larger groups, and/or were less isolated from one another.

However, the allelic identities show an even more pronounced relationship to the coastal/inland residential distinction. Without exception, the coastally-located populations have lower allelic identities than the inland populations. Two of the coastally-located Papuan-speaking groups (Sulka and Kuot) fall in this lower allelic identity coastal grouping, and two of the inland Oceanic-speaking groups (Mamusi and Nakanai-S) fall in the higher allelic identity interior grouping. These linguistic “outlier” populations probably reflect recent population movements between the New Britain coast and interior.

### The Branching Model

**Figure 3:**
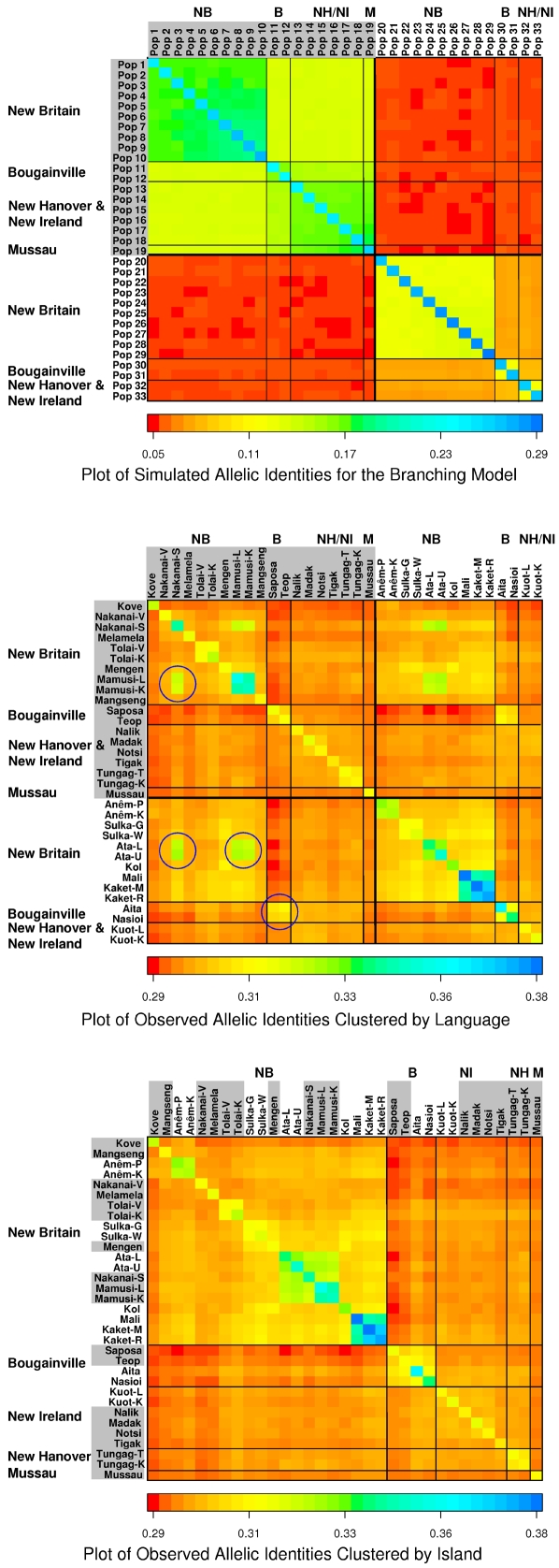
Simulated and observed heat plots for the branching model. The heat plots are color-coded representations of the square matrix of within- and between-population allelic identities. The level of allelic identity is indicated by the color-scale at the bottom of each plot. The diagonals represent the within population allelic identities, and the off-diagonals represent the between-population identities. Population names are located above and to the left of the matrix. The Oceanic-speaking populations are shaded in gray. (A,B) The populations are clustered first by language group, then by island. (B) The circled population groupings have high allelic identity even though the populations are in different language groups. (C) The populations are clustered only by island and neighborhood.

As mentioned, Figure 2 shows the presumed history of population splits used as the basis for the simulated branching model. Figure 3A shows the simulated heat plot derived from the simulations of this branching history. The simulated allelic identities in Figure 3A are lowest between the Oceanic and Papuan populations, higher between populations on different islands, higher still between populations within islands, and highest within populations. The level of allelic identity is also uniform between populations at different levels in the hierarchy, reflecting the isolation of branches following ancient population splits. The hierarchical organization and the uniformity of allelic identity within major clusters are fundamental properties of the branching process.

Figure 3B shows the observed allelic identity heat plot, with the populations arranged in the same order as in 3A (i.e., clustered first by language group, then by island). The poor fit with the predicted properties of the branching model in 3A is obvious. The Oceanic-Papuan comparisons do not have low and uniform allelic identities. For example, the allelic identities between the Oceanic-speaking Mamusi and Nakanai-S on the one hand and the Papuan-speaking Ata on the other are high compared to the identities between same-language-speaking populations (Figure 3B, circled squares). These are three neighboring groups in the interior of central New Britain. Identities are also high between the four Bougainville populations, even though two of them speak Oceanic languages (Saposa and Teop) and two speak Papuan languages (Aita and Nasioi).

Figure 3C shows the same allelic identities arranged simply by island and neighborhood (i.e., not by language). While the fit to the expected pattern is still poor, this reordering shows that allelic identities are relatively high between populations on the same island, and relatively low and uniform between populations on different islands. It also underlines the high identities between the linguistically diverse Mamusi, Nakanai-S, and Ata in the New Britain interior, and between the different language speaking populations on Bougainville.

In sum, the observed pattern of allelic identity variation is not consistent with the branching model. It shows that significant genetic exchange has occurred between local populations within islands whether they belong to the same major language group or not, but that genetic exchange between islands may have been relatively restricted for some time.

**Figure 4:**
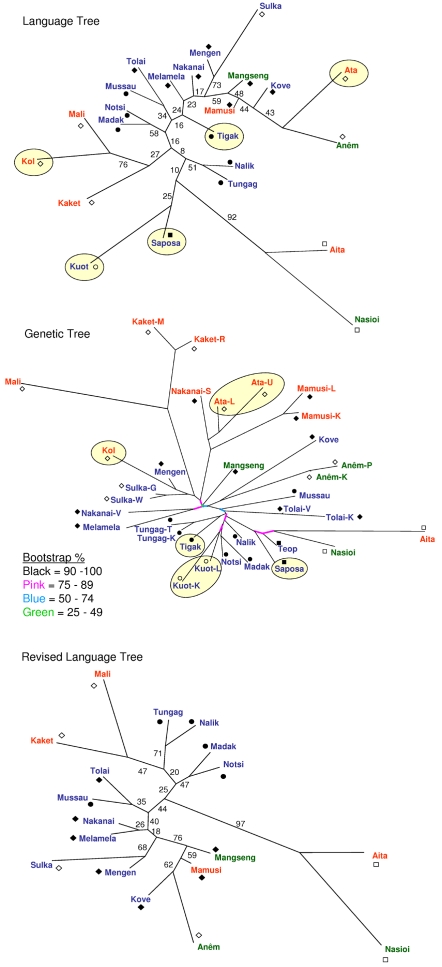
Genetic and language trees. (A) Language tree. (B) Genetic tree. (C) Revised language tree after removing outliers. The symbols and colors associated with the population names are the same as those used in Figure 1. Bootstrap values for the language tree and revised language tree are listed next to each branch. Because there was insufficient room to list the numeric values next to many of the small branches in the genetic tree, bootstrap values in those cases are indicated by the branch color. The outlier populations identified from the observed vs. expected genetic distance plots (Figure 5) are highlighted with yellow circles. These populations are absent from the revised language tree. The genetic tree contains more populations than the language tree because biological samples were collected from several populations that spoke the same language (e.g., the genetic sample contains two Anêm-speaking populations).

The language and genetic trees in Figure 4 reinforce this scenario. Neither tree completely separates the Oceanic- from the Papuan-speaking populations. Instead, the trees tend to group populations from the same island. The island grouping is particularly strong for the genetic tree, which also clusters geographic neighbors within islands better than the language tree, e.g., it contains the Mamusi/Nakanai-S/Ata cluster from inland New Britain. The language tree does not contain this cluster, but instead groups the geographically distant Ata and Anêm together, both of which speak Papuan languages. Overall, the language tree has a stronger tendency than the genetic tree to group Papuan-speaking populations separately from Oceanic-speaking populations, suggesting that structural linguistic features are more resistant to exchange than genes between the major language groups, or that linguistic exchange has been comparatively more common within the language groups than between them. The results may also reflect relatively low information content in the linguistic data. The bootstrap values of the language tree are low, and the linguistic data contain only 108 features compared to the 6,437 alleles for the microsatellite loci.

**Figure 5:**
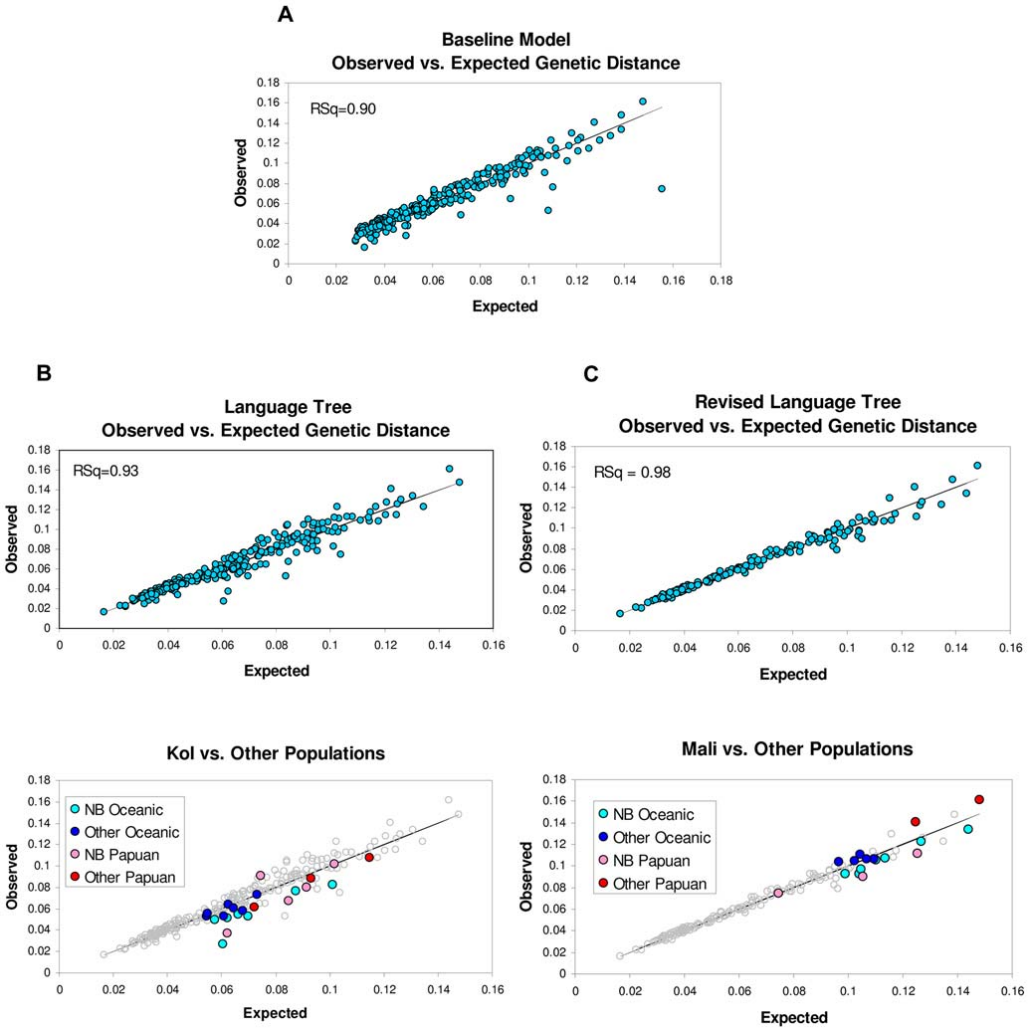
Plots of observed distances estimated from the microsatellites vs. the expected genetic distances estimated for the baseline and language trees. R-squared values are indicated on the plots. (A) Comparison of the observed genetic distances with predicted distances for the baseline tree. (B) Comparison of the observed genetic distances with predicted distances for the language tree. The bottom figure highlights the Kol vs. other population comparisons. (C) Comparison of the observed genetic distances with predicted distances for the revised language tree (outlier populations removed). The bottom figure highlights the Mali vs. other population comparisons.

The results of the model-fitting procedure are shown in Tables 2 and 3. The Λ values for the fitted baseline and language trees are reported in Table 2. Λ for the baseline tree is very high relative to the degrees of freedom, indicating that it does not capture the genetic structure of the NIM populations very well. The lack of fit is also shown by the plot of the observed genetic distances vs. the expected genetic distances for the baseline tree shown in Figure 5A. This result is not surprising given the lack of similarity between the structure-less baseline tree and the topologically complex genetic tree. However, even though the observed and expected genetic distances are not perfectly congruent, the correlation coefficient for the plot is fairly high, indicating that even the baseline tree captures some of the genetic structure of NIM populations. The reason for the high correlation is that the model-fitting procedure estimates the individual population allelic identities fairly accurately for the baseline tree, and this identity is one of the two parameters used to estimate genetic distance. The reason the correlation is not even higher is that the other parameter used to estimate genetic distance is the between-population allelic identity, and, since the baseline tree has only one internal node, the model-fitting procedure estimates only one value for this between-population identity. In the observed data, there are many different values for the between-population identities, causing the discrepant results.

**Table 2:** Λ values for the baseline and language trees.

Model	Λ	df
Baseline tree	18078	252
Language tree	8593	231
Tips only language tree	14305	244
Revised language tree	1992	136

**Table 3:** Comparison of fit of various models to the baseline and language trees.

Model	Reduction in Λ vs baseline tree	F-ratio	P-value
Language tree	9484	1.93	0.0000
Tips only language tree	3773	1.22	0.0566
Revised language tree	16086	4.90	0.0000
	**Reduction in Λ vs language tree**		
Revised language tree	6601	2.54	0.0000

Λ is much lower for the fitted language tree than it is for the fitted baseline tree (Table 2). The F-test indicates that the superior fit is statistically significant (Table 3). This superior fit may not be because of any deep congruence between the linguistic and genetic structures, but only because of a few superficial internal nodes (tips) shared by the language and genetic trees (e.g., Aita - Nasioi). To test this possibility, we used the model-fitting method to fit a tree that contained only these shared tips. Λ for this tips-only tree was much lower than it was for the baseline tree (Table 2), but it was still not nearly as low as it was for the complete language tree. This result suggests that the language tree captures more than just some superficial aspects of the genetic structure.

Figure 5B is the plot of the observed genetic distances vs. the expected genetic distances based on the language tree. The relatively high squared correlation for the plot also confirms that the language tree captures more of the genetic structure than the baseline tree. There are, however, several clear outlier points in the plot, and Λ is still very high for the language tree relative to its degrees of freedom, meaning that its fit is far from perfect.

The lower plot in Figure 5B shows that of all of the groups, the Kol contribute most to the high Λ of the language tree. Λ for the language tree reconstructed after removing the Kol is 5,777 compared to 8,593 for the full language tree (see Table 4). The plot shows that the Kol are generally closer to neighboring populations than the language tree would predict, reflecting the greater tendency of the genetic tree to group neighboring populations on the same island. For example, in the genetic tree, the Kol, who speak a Papuan language, cluster with the nearby Oceanic-speaking Mengen, whereas in the language tree, they cluster with other Papuan-speaking populations who are more distant geographically. These different tree patterns confirm the greater tendency of genes to move between Papuan- and Oceanic-speaking populations than structural linguistic features.

**Table 4:** Reduction in model Λ after sequential removal of major outlier populations.

Model	Λ	df	Reduction in Λ compared to previous model^a^
Full Model	8593	231	
**Population removed**			
Kol	5777	210	2816
Ata	4242	190	1535
Kuot	3292	171	950
Saposa	2434	153	858
Tigak	2108	136	326
Sulka	1677	120	431
Mengen	1319	105	358
Nasioi	1075	91	244
Notsi	777	78	298
Mangseng	656	66	121
Nalik	542	55	114
Aita	359	45	183
Mali	294	36	65
Kaket	203	28	91
Mussau	117	21	86
Tolai	51	15	66

a See Text S1.

The contributions of other populations to the lack of correspondence between the observed and expected genetic distances are shown in Table 4. Methods described in Text S1 were used to identify four additional populations that contributed disproportionately to the lack of correspondence. Three of these four outliers also involved neighboring Oceanic- and Papuan-speaking populations that clustered together in the genetic tree but not in the language tree. Λ for the language tree lacking the Kol and these other four outlier populations is 1,992 (Table 2), which represents a dramatic reduction compared to the full 23 population language tree (F-test p<0.0001, Table 4).

The revised 18-population language tree is shown in Figure 4C, and the plot of the observed genetic distances vs. the expected genetic distances for this revised tree is shown in Figure 5C. The very high squared correlation coefficient in 5C confirms its superior fit relative to the full 23-population language tree. However, Λ is still high for this revised language tree, indicating that even it does not fully capture the genetic structure of NIM populations. The lower plot in Figure 5C shows that the Mali are the largest outlier in this comparison. The Mali are closer to other New Britain populations in the genetic tree, regardless of the language they speak, than they are in the language tree. Overall, the results show the pervasive pattern of closer genetic than linguistic proximity between populations on the same island.

### The Isolation by Distance Model

**Figure 6:**
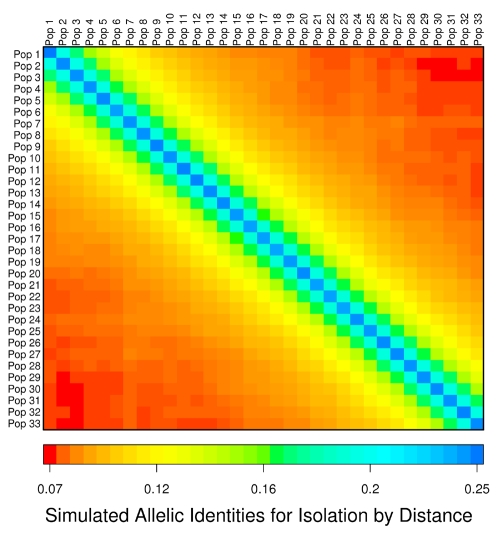
Simulated isolation by distance heat plot. Additional isolation by distance results are shown in Text S1.

Figure 6 shows the heat plot for the simulated isolation by distance model allelic identities. The simulated identities are highest within populations and then fall off steadily as the geographic distance between populations increases (indicated by the change in color moving horizontally or vertically away from the diagonal). There is some hint of this fall-off for some populations in the observed matrix, but, overall, the observed pattern diverges from the predicted.

In the simulations, the populations are arrayed next to one another in a linear stepping stone pattern, but the 33 sampled NIM populations are not located next to one another in a simple linear fashion. However, the lack of congruence between the heat plots is not because of this difference. Isolation by distance predicts decreasing allelic identity with increasing geographic distance regardless of the actual sampling locations, and this pattern does not occur for the observed allelic identities. This conclusion is supported by additional simulations reported in the last section of Text S1.

Table 5 shows the matrix correlation results. Waypoints did not improve the correlations, so we report only the results for the direct great circle distances. The correlations listed for the full sample are suggestive of an isolation by distance coevolutionary process in the region, but several of the correlations are not statistically significant at the multiple tests-adjusted level. However, when the correlation coefficients are calculated for localized geographic and linguistic comparisons, many of them increase in magnitude and cross the threshold of statistical significance.

**Table 5:** Correlations of genetic, linguistic and geographic distances in the full sample and in localized geographic and linguistic subsets.

	Genetic-geographic	Linguistic-geographic	Linguistic-genetic	Gen-ling partial correlation
	r	r	r	r
Full Sample	0.31*	0.29*	0.49**	0.44**
Interior	0.62*	0.64**	0.75**	0.58**
Coast	0.54**	−0.01	0.30	0.36**
Papuan	0.45	0.52**	0.60**	0.47**
Oceanic	0.26	0.40**	0.05	−0.06
New Britain - All populations	0.16	0.25	0.44**	0.42**
New Britain - Interior only	0.94**	0.59**	0.67**	0.41
New Britain - Coastal only	0.64	0.39	0.55**	0.43
New Ireland & New Hanover	0.43	0.09	0.37	0.37

*: p<0.005.

**: Sig. at multiple tests adjusted p = 0.0024.

**Figure 7:**
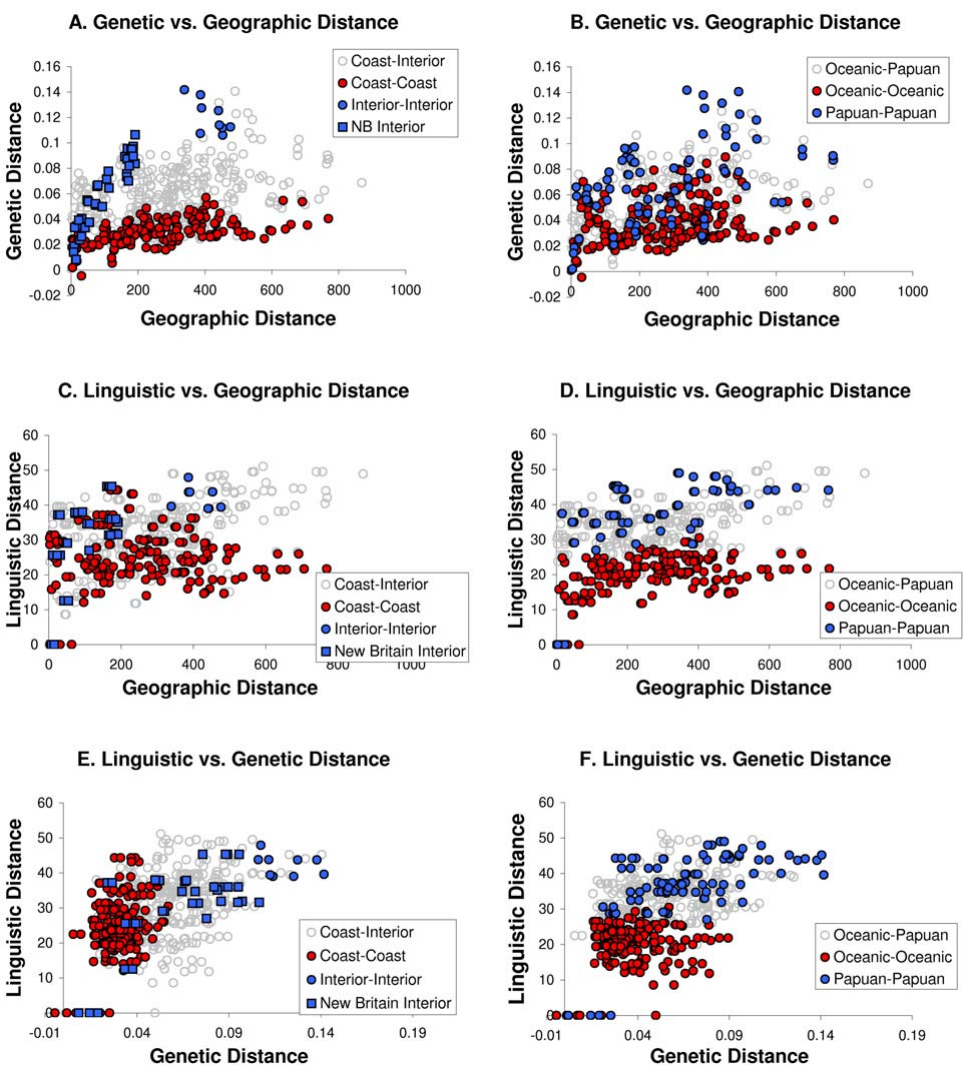
Plots of genetic, linguistic, and geographic distance comparisons. Coastal vs. coastal (red circles) and interior vs. interior (blue circles) are highlighted in the plots on the left. Blue squares highlight the interior New Britain comparisons. Oceanic vs. Oceanic (red circles) and Papuan vs. Papuan (blue circles) comparisons are highlighted in the plots on the right. (A,B) Genetic vs. geographic distance plots. (C,D) Linguistic vs. geographic distance plots. (E,F) Linguistic vs. genetic distance plots.

Figure 7 shows plots of the genetic, linguistic and geographic correlations and highlights the localized geographic and linguistic comparisons. Figure 7A and 7B shows the genetic-geographic distance correlation, with different localized sets highlighted. In Figure 7A, the interior and coastal sets are highlighted in red and blue. The lack of mixing of the colors suggests that there has been limited genetic exchange between island interiors and coasts. Figure 7B highlights the Papuan and Oceanic sets. The mixing of the colors shows that Papuan and Oceanic-speaking populations have exchanged genes. This exchange has occurred primarily between the interior Oceanic-speaking Mamusi and Nakanai-S with interior Papuan-speaking populations, and between the coastal Papuan-speaking Kuot and Sulka with coastal Oceanic-speaking populations. Table 6 shows how the Oceanic and Papuan genetic-geographic distance correlations improve when these four outlier populations are removed.

**Table 6:** Correlations of genetic, linguistic, and geographic distances with interior Oceanic- and Coastal Papuan-speaking populations removed.

	Genetic-geographic	Linguistic-geographic	Linguistic-genetic	Gen-ling partial correlation
	r	r	r	r
Full Sample	0.27	**0.32** **	**0.68** **	**0.65** **
Interior	0.59	**0.74** **	**0.78** **	**0.63** *
Coast	0.54**	**0.26**	**0.40** **	0.32*
Papuan	**0.60** **	**0.62** **	**0.77** **	**0.63** **
Oceanic	**0.54** **	0.29**	**0.41** **	**0.32** **
New Britain - All populations	**0.20**	0.24	**0.68** **	**0.67** **
New Britain - Interior only	0.94**	**0.76**	**0.84** *	**0.54**
New Britain - Coastal only	0.64	**0.53**	**0.83** **	**0.76** **
New Ireland & New Hanover	**0.51**	0.09	**0.50**	**0.52**

*: p<0.005.

**: Sig. at multiple tests adjusted p = 0.0024.

Increased correlations compared to Table 5 are highlighted in bold text.

Plots 7C and 7D show the linguistic-geographic distance correlations, with the different sets highlighted as before. As one might expect for the linguistic correlations, the coastal and interior strata are less clearly distinguished than the Oceanic and Papuan strata. This is again consistent with the argument that there has been little linguistic exchange between Oceanic and Papuan languages where they occur in neighboring groups (e.g., the four outliers). The poorer distinction for the interior and coastal strata is caused by these outliers. Table 6 shows that the interior and coastal linguistic-geographic distance correlations improve dramatically when the four outliers are removed.

Plots 7E and 7F show the genetic-linguistic distance correlations with similar highlighting. They suggest that any linguistic-genetic correlation is driven solely by the Papuan-speaking populations, but as Table 6 shows, when the four outliers are removed, the correlation for the Oceanic comparisons increases dramatically and becomes statistically significant. These results provide further support for the conclusion that linguistic exchange has been comparatively limited between Oceanic- and Papuan-speaking populations where they overlap geographically.

The plots also show that for any given geographic distance, the interior/Papuan-speaking populations have higher genetic and linguistic distances among them than do the coastal/Oceanic-speaking populations. The correlation coefficients are also generally larger between interior/Papuan populations than they are between coastal/Oceanic populations. This distinction is the result of the comparatively restricted movement in the rugged highland interiors [68], coupled with the much longer tenure of Papuan-speaking populations.

The correlations are particularly high in the New Britain interior (Table 5, blue squares in Figure 7). The genetic-geographic distance correlation is 0.94 (p<0.0000), which, to our knowledge, is the highest such correlation reported for any region worldwide. The high linguistic-geographic (0.59) and genetic-linguistic correlations (0.67) for the New Britain interior are also significant at a high level of probability, but the partial correlation, in which geographic distance is held constant, is not. As mentioned, the correlation and partial correlation patterns are consistent with an isolation by distance process where genetic and linguistic exchange have occurred largely independently of one another.

The results on the New Britain coast suggest a separate isolation by distance pattern there as well. All of the correlation coefficients there are high, but only the genetic-linguistic distance correlation is statistically significant (Table 5). The p-values for the other correlations are low (genetic-geographic = 0.0066; linguistic-geographic = 0.0099), but they are above the multiple tests adjusted significance level (p = 0.0024). When the two Papuan-speaking populations are removed from the coastal New Britain sample, the correlations increase in magnitude and the partial correlation also crosses the threshold of statistical significance (Table 6), despite the fact that the sample contains only six populations. We suspect that a larger sample would reveal an even more robust isolation by distance pattern on the coast and on the other islands in the region.

## Discussion

### Branching versus Isolation by Distance Coevolution

The tests of the branching model in Northern Island Melanesia show that genetic and linguistic exchange between local populations has erased evidence that may have once existed for a branching process there. Genes have tended to move freely between nearby populations, regardless of the languages they speak. On the other hand, structural linguistic exchange has been particularly limited between neighboring Oceanic and Papuan languages. In these instances, the Oceanic-speaking populations have become very similar genetically to their Papuan-speaking neighbors (the best example of this is the high allelic identity between the Ata, Mamusi and Nakanai-S shown in the heat plot in Figure 3B). Although an alternate explanation for this situation is that Oceanic languages have simply been adopted by formerly Papuan-speaking groups [c.f., 50], this now appears most unlikely, because the general tendency in Northern Island Melanesia is for neighboring populations, regardless of their languages, to become genetically similar (other clear examples are the Kove/Anêm and also the Kuot and their neighbors on New Ireland). Previous analyses of the autosomal microsatellites [50] as well as Y-chromosome data [67] suggest that Papuan-speaking groups, who entered NIM first and expanded there long before the arrival of the early Oceanic-speakers, have contributed much more genetically to Oceanic-speaking groups than vice versa over the last three millennia.

The genetic, linguistic and geographic distance correlations are consistent with an isolation by distance coevolutionary process in the interior of the largest island in the region, New Britain. For the correlations to be so strong, the patterns of ancestral residence and local migration must have persisted for a considerable period. It is remarkable that the patterns have persisted in the face of the destabilizing influence of European contact [42],[69] and also of displacements caused by major volcanic eruptions [70]. One reason for the persistence is the continuing ties of the people to their land. Even today, most people in our sample remain in small villages and continue to farm their local gardens, or they maintain dual residences there and in larger population centers [68].

The matrix correlation results show that studies of prehistory and coevolution at the regional level must take into account the geographic and linguistic heterogeneity of a region, since ecological and sociocultural variation are likely to strongly influence biological and cultural patterning. Parallels to the heterogeneity found in NIM probably exist, in many cases unidentified, in every major world region and in various locations within each region [71]–[74].

### Coevolution at Larger Geographic Scales

Our results are apparently at odds with the studies of Cavalli-Sforza et al. [4],[5] that identified a strong correspondence between global gene and language trees. One explanation is that global patterns are more likely to emphasize ancient demographic events, such as population splits associated with the colonization of major world regions, while local patterns will generally emphasize more recent demographic events. Wilkins and Marlowe [75], for example, showed that genetic data collected from local populations are more likely to reveal recent changes in migration associated with the rise of agriculture than data collected from a global sample. However, it is also possible that the differences between the global results of Cavalli-Sforza and colleagues and ours are not so pronounced. In their studies, they identified several instances of disagreement between the language and genetic trees caused by different patterns of genetic and linguistic exchange and language shift, so the global pattern may also reflect, to a substantial degree, the types of local population interactions we identified in NIM.

### The Importance of Highly Informative Datasets

The structural linguistic data used in this study [48],[76] have recently come under attack, both in terms of their quality and what they capture (i.e., just more recent contacts, or mainly ancient language splits). Our results certainly suggest that structural features may well be more resistant to dynamics of diffusion than genes, and therefore likely contain considerable information about language splits as well as language contacts. The structural features may also be more resistant to diffusion than lexical items, making them more suitable than cognate data for examining linguistic splits in NIM, and probably in other regions as well.

Dunn et al. [48],[49] have addressed the criticisms of data quality in detail, but they acknowledge that there are some problems. The linguistic features are not completely independent of one another, the data may contain substantial homoplasy [37],[49], and for the NIM dataset, there are 8.7% missing data. Despite these shortcomings, the significant correlations between the linguistic, genetic, and geographic distances certainly show that the structural linguistic data contain important information about the relationships between NIM languages. In particular, the separation of the Oceanic and Papuan groupings in the plots of linguistic vs. geographic distances (Figure 7D) suggests that, even if the data *only* reveal linguistic contacts, the contacts have been stronger between populations within each major language group than between populations in different language groups [see also 39].

Another relevant point is that the linguistic data and methods typically used in studies of coevolution have usually been of comparatively poor quality. To illustrate the higher quality of our structural linguistic dataset, we employed the commonly used method of node counting to estimate linguistic distances between NIM languages in a classification constructed using the Ethnologue (http://www.ethnologue.com/), and we then examined the correlation between these distances and the genetic and geographic distances. None of the correlations were statistically significant. If not for the structural linguistic data, we would have failed to identify any linguistic relationship to genetic or geographic patterns at all.

The limitations of these sorts of data are not restricted to Northern Island Melanesia. Hunley et al. [16] tested the branching and isolation by distance models in South America, where linguistic divergence has been occurring for a considerably shorter period. They examined the fit of language and gene trees constructed from linguistic cognate data and mtDNA sequences, and identified correspondences only between the tips of the language and genetic trees, i.e., only between very recently diverged groups. In the current study, the language and genetic structures shared more than just a few superficial similarities, clearly suggesting the results are indicative of more ancient relationships. Studies of coevolution will clearly benefit greatly from using similar structural linguistic datasets.

The highly informative nature of the genetic data available to us (i.e., the 751 microsatellite loci with 6,437 different alleles) also undoubtedly led to our finding of comparatively high correlations in our various analyses. Many recent studies have used mitochondrial d-loop data and Y-chromosome data to investigate genetic and linguistic correspondence in various world regions [15], [16], [20], [77]–[81], but these data are comparatively uninformative. The Y-chromosome data typically contain only a few loci, and the mitochondrial d-loop data are plagued by homoplasy, which confounds the construction of genetic classifications and limits the accuracy of genetic distance estimation [82]. In an earlier publication, information content issues prevented us from successfully fitting our structural language tree to mtDNA and Y-chromosome data collected from most of the same populations [66]. The mitochondrial d-loop data were able to recreate some of the same correlation patterns we found using the autosomal microsatellite data, but the correlations were always weaker than those we have reported here.

### Implications for Pacific Prehistory

The implications of our results for broader issues in Pacific prehistory are important but must be interpreted carefully. While our results provide little support for the branching model in Northern Island Melanesia, this is different from arguing that branching did not occur in very early periods there, or elsewhere in the Pacific, and it does not mean that our microsatellite data lack important information about the deeper prehistory of the entire region.

For example, two contrasting scenarios for the origins of the Polynesians have persisted in recent Pacific prehistory debates, and they bear a very close relationship to the two models examined in this paper. The first has been called the phylogenetic model [83],[84], which is essentially identical to the branching model, and the second, called a reticulate model [85], is essentially identical to the isolation by distance model [see also rebuttal by 86]. A number of mixed models, perhaps more realistic than either of these, have also been proposed [87]. Bellwood [83] also argued that phylogenetic differentiation should be expected to occur primarily during or shortly after the early rapid range expansions in new territories, while the reticulate model, which stresses a continuous and relatively uncoordinated shifting of linguistic, cultural, and biological boundaries through assimilation, intermarriage, borrowing, and diffusion, may become more evident in subsequent periods.

The genetic data have been interpreted to support several of these Polynesian origin scenarios. Some have indicated that a clear phylogenetic signal exists between Taiwan Aborigines and Polynesians, with little intermixture taking place in Near Oceania, while other datasets have been interpreted to suggest heavy intermixture with, or major contributions from, Near Oceanic and Wallacean populations [50], [65], [88]–[93]. While the results of our present study are broadly inconsistent with phylogenetic models in Northern Island Melanesia, our group did identify in the same microsatellite data a small but clear genetic coancestry between certain Taiwanese populations and Oceanic-speaking groups in Island Melanesia, as well as a much stronger Taiwan Aboriginal signal in Polynesia, indicating that intermixture over the past 3,000 years has not completely erased genetic signals of early Oceanic origins in either NIM or Polynesia [50]. The more comprehensive nature of our genetic and linguistic coverage in this region has now allowed a more complete, if complex, picture of ancient population dynamics to emerge.

## Supporting Information

Text S1 Supplemental materials and methods.
(0.09 MB DOC)
