# Supplementary material for: Genetic and Linguistic Coevolution in Northern Island Melanesia
Source: PLoS Genet. 2008 Oct 31;4(10):e1000239. doi: 10.1371/journal.pgen.1000239 (PMC2570610; doi:10.1371/journal.pgen.1000239)
Supplement: Text S1 — Supplemental materials and methods. (0.09 MB DOC) [file pgen.1000239.s001.doc]

TEXT S1

COALESCENT SIMULATIONS. We used the computer package Simcoal [v2.1, 1] to simulate the branching and isolation by distance models. For the branching model, we simulated the population history depicted in Figure 2 (in the main text). Each population was comprised of multiple diploid genomes, and each genome was comprised of multiple unlinked microsatellite loci. The loci accumulated mutations under a stepwise model [2] at a rate of 0.0075 mutations per generation [3]. Moving backwards in time, the populations merged in the pattern depicted in Figure 2. The first mergers occurred between populations within islands beginning 75 generations before the present (bp). The ancestral island populations merged in a nested fashion between 150 and 2000 generations bp, with the final merger occurring 4000 generations bp, representing the initial population split in Asia. Other population branching times and branching patterns were also simulated. Their common thread was the deep split between the Oceanic and Papuan branches and the absence of migration between populations. The reported allelic identity pattern was unaffected by these adjustments or by the adjustment of the number of genomes per population, the number of unlinked loci per genome, or population growth rates.

For the isolation by distance simulations, populations fused into a single ancestral population at a single time in the past. Populations were arrayed end to end in a linear stepping-stone fashion, and, prior to fusion, each population exchanged a constant portion of its genomes each generation with its immediate neighbors. In different replicates of the simulations, we varied the number of genomes/population, the number of loci/genome, the fusion time, and the proportion of migrants/population/generation. Varying these parameters did not affect the reported allelic identity pattern.

We also constructed an isolation by distance model in which populations exchanged genomes with geographic neighbors in two dimensions. In the 2-dimensional simulations, we used a 7 x 7 grid of 49 populations. Both the linear and 2-dimensional models showed a steady decay in allelic identity with increasing geographic distance between populations. We report only the linear stepping-stone results in the main text.

Fifty simulations sufficiently captured the pattern of allelic identity variation associated with each model. Following each simulation, twenty diploid genomes were sampled from each population, from which the within- and between-population allelic identities were estimated. The allelic identities were then averaged for the 50 simulations and plotted in the color-coded heat plots.

TREE CONSTRUCTION. The language trees were estimated using two simultaneous, completely independent, analyses starting from different random trees, using the Mr. Bayes v3.1 [4] computer package. The analyses were continued until the standard deviation of the split frequencies between the runs was less than 0.01. We used the program defaults for “standard” data for the prior probability distribution for the parameters of the likelihood model. The defaults included: 1) all possible trees were considered *a priori* to be equally probably; 2) the probability of a change from one binary linguistic feature state to the other was equal; 3) unconstrained branch lengths with uniform prior probabilities. After discarding the initial 250 trees generated by the program, approximately 1000 trees were retained, and the reported bootstrap values for the language tree and revised language tree represent the percent of time a particular branch was observed in the 1000 or so trees. All estimated branch lengths were significantly greater than zero. Language trees were also constructed using distance-based and parsimony methods, and the tree topologies and the model fitting results and conclusions were similar for all construction methods.

The gene tree was constructed from R_ST_ genetic distances [2] obtained using Arlequin v3.1 [5]. Trees were also constructed from F_ST_-based distances [using the transformation of 6] and from minimum genetic distances [7]. All trees had nearly identical topologies. The bootstrap values were obtained from microsatellite allele frequencies estimated using CONVERT [8] and the PHYLIP package [9]. Trees were drawn using TREEVIEW [10] and modified by hand.

OUTLIER POPULATION IDENTIFICATION IN THE OBSERVED VS. EXPECTED GENETIC DISTANCE PLOTS. To identify the populations that contributed most to the lack of fit of the language tree, we removed the linguistic data for one population, made a new language tree (using the Bayesian approach), and then refit the new tree. This procedure was implemented 23 times, once for each population. The procedure indicated that, by a wide margin, the Kol contributed most to the lack of fit of the language tree. We then repeated the procedure 22 times for the Kol-less tree. The population that contributed most to the lack of fit was then removed, a new tree was made, and the model-fitting procedure was applied to the new tree. This procedure was repeated until the reduction in Λ leveled off, which occurred after an additional 4 populations were removed (see Figure S1). The 5 removed populations were the Kol, Ata, Kuot, Saposa and Tigak (see also Table 4 in the main text).

FIGURE S1

LINGUISTIC DATA AND SUPPLEMENTAL ANALYSES. Table S1 is the matrix of linguistic distances. Figure S2 shows the distribution of the linguistic distances in localized geographic and linguistic subsets of the data. The top plot highlights the large linguistic distances among the Papuan languages compared to Oceanic languages. The distances between the Oceanic and Papuan languages are about as large as those between Papuan languages, though some of the distances are more intermediate between the two groups, which is consistent with previous findings that there has been some structural linguistic exchange between the language groups [11,12].

The bottom plot highlights the linguistic distances for the coastal and interior sets. The similar distributions to the top plot reflect the strong association between language and location, i.e., Oceanic languages are located mainly on the coasts, and Papuan languages are located mainly in island interiors. The lowest inter-location values (the three leftmost yellow bars) are between the Oceanic languages located in the interior of New Britain (Mamusi and Nakanai-S) and the Oceanic languages on the coasts of the island. The distances are typical of those between other Oceanic languages (top plot). In contrast to the linguistic pattern, the allelic identity in the Mamusi and Nakanai-S is high (see main text and Table 1), which is more typical of Papuan-speaking groups. The Oceanic-typical linguistic distances but high allelic identity for the two populations may reflect a genetic founder effect that occurred when the Mamusi and Nakanai-S moved from the New Britain coasts to its interior.

FIGURE S2

HEAT PLOT OF NEW BRITAIN INTERIOR. In the isolation by distance simulations, the populations are arrayed next to one another in a linear stepping stone, but the 33 sampled NIM populations are not located next to one another in a simple linear fashion. Figure S3 shows why the lack of congruence between the observed heat plot and the simulated isolation by distance heat plot is not the result of this lack of geographic contiguity of the NIM populations. Figure S3A is the heat plot for 9 contiguous populations from an isolation by distance simulation of 50 populations, and Figure S3B is the observed heat plot for the 9 interior New Britain populations. The plots look different because the 9 simulated populations are geographically contiguous, while the 9 observed populations are from very different geographic locations in the New Britain interior. Five of them are located close to one another in central New Britain, another is located in the central eastern portion of the island and three are located in the north. Figure S3C contains 9 populations from the isolation by distance simulations sampled in 3 non-contiguous blocks: 5 contiguous populations, a geographic gap of 12 populations, 1 population, a geographic gap of 3 populations, and 3 contiguous populations. The simulated between-group allelic identity pattern in Figure S3C is remarkably similar to the observed pattern in Figure S3B. Both plots capture the allelic identity gaps between the non-contiguous blocks, caused by the gaps in geographic distance, and both plots show a steady decrease in allelic identity away from the diagonals, representing a steady decay in genetic similarity with increasing geographic distance.

FIGURE S3

LITERATURE CITED

1. Laval G, Excoffier L (2004) SIMCOAL 2.0: a program to simulate genomic diversity over large recombining regions in a subdivided population with a complex history. Bioinformatics 20: 2485-2487.

2. Slatkin M (1995) A measure of population subdivision based on microsatellite allele frequencies. Genetics 139: 457-462.

3. Brinkmann B, Klintschar m, Neuhuber F, Huhne J, Rolf B (1998) Mutation rate in human microsatellites: influences of the structure and length of tandem repeat. American Journal of Human Genetics 62: 1408-1415.

4. Huelsenbeck JP, Ronquist F (2001) MRBAYES: Bayesian inference of phylogeny. Bioinformatics 754-755.

5. Excoffier L, Laval G, Schneider S (2005) Arlequin ver. 3.0: An integrated software package for population genetics data analysis. Evolutionary Bioinformatics Online. pp. 47-50.

6. Reynolds J, Weir BS, Cockerham CC (1983) Estimation of the Coancestry Coefficient: Basis for a Short-Term Genetic Distance. Genetics 105: 767-779.

7. Nei M (1987) Molecular Evolutionary Genetics. New York: Columbia University Press.

8. Glaubitz JC (2004) CONVERT: A user-friendly program to reformat diploid genotypic data for commonly used population genetic software packages. Molecular Ecology Notes 4: 309-310.

9. Felsenstein J (2005) PHYLIP (Phylogeny Inference Package) version 3.6. Seattle: Distributed by the author. Department of Genome Sciences, University of Washington.

10. Page R (1996) TREEVIEW: An application to display phylogenetic trees on personal computers. Computer Applications in the Biosciences 12: 357-358.

11. Lindström E, Terrill A, Reesink G, Dunn DM (2007) The Languages of Island Melanesia. In: Friedlaender JS, editor. Genes, Language, and Culture Change in the Southwest Pacific. New York: Oxford University Press. pp. 118-140.

12. Reesink G (2005) Sulka of East New Britain: a mixture of Oceanic and Papuan traits. Oceanic Linguistics 44: 145-193.
